# Disparities and outcomes of patients living with Down Syndrome undergoing healthcare transitions from pediatric to adult care: A scoping review

**DOI:** 10.1002/ajmg.a.62854

**Published:** 2022-06-10

**Authors:** Karan Varshney, Rosemary Iriowen, Kayla Morrell, Preshon Pillay, Alexander Fossi, Mary M. Stephens

**Affiliations:** ^1^ Jefferson College of Population Health Thomas Jefferson University Philadelphia Pennsylvania USA; ^2^ School of Medicine Deakin University Geelong Victoria Australia; ^3^ Sidney Kimmel Medical College Thomas Jefferson University Philadelphia Pennsylvania USA; ^4^ Center for Autism and Neurodiversity Thomas Jefferson University Philadelphia Pennsylvania USA; ^5^ Department of Family & Community Medicine Thomas Jefferson University Philadelphia Pennsylvania USA; ^6^ Center for Special Healthcare Needs Christiana Delaware USA

**Keywords:** disparities, Down Syndrome, equity, healthcare transition, pediatrics

## Abstract

Down Syndrome (DS) is one of the most common chromosomal disorders worldwide, and people with DS experience more co‐morbidities and have poorer health outcomes compared to the general population. An area that is not well understood is how patients with DS transition from pediatric to adult care, as well as the details, barriers, and difficulties of these transitions for patients. Hence, we aimed to provide a scoping review of the literature in PubMed, Scopus, and CINAHL on the topic of healthcare transitions (HCTs) for patients with DS. Findings suggest patients with DS who continued receiving care as an adult from a pediatric care provider tended to experience co‐morbidities and other adverse health issues at higher rates than those who entirely switch to an adult‐care team. Patients with DS were unable to undergo transition due to multiple barriers, such as low income, limited/public insurance, gender, and race. We propose potential steps for transition, which focus on ensuring early planning, communicating better, coordinating services, assessing decision‐making capacity, and providing ongoing social and financial support. Future research must further identify and address barriers to HCTs for people with DS.

## 1 INTRODUCTION

Down Syndrome (DS) is a chromosomal disorder that is prevalent worldwide. Though estimates vary, it is believed that the incidence of DS is 1 in 1000 to 1 in 1100 births (United Nations, 2021). However, regional variation has been shown in the prevalence and incidence of DS. For example, in England, the live birth prevalence of DS is 12.3 (11.6–12.7) cases per 1000 live births (Doidge et al., 2020), whereas, in China, the DS total prevalence ratio from 2003 to 2011 was shown to be 3.05 per 10,000 births (Deng et al., 2015). In the United States, DS is currently the most common chromosomal disorder with approximately 6000 babies born with DS every year (Center for Disease Control and Prevention, 2020); some estimates suggest that the population prevalence of DS in the United States will continue increasing with time, while others suggest that it is at a plateau (Bittles & Glasson, 2004; de Graaf et al., 2017).

Since overall life expectancy is expected to increase as a result of medical improvements and increased support (Bittles & Glasson, 2004; Center for Disease Control and Prevention, 2021), those living with DS likely will also see a rise in life expectancy in the future (Centers for Disease Control and Prevention, 2021). However, as people with DS live longer, physicians and healthcare providers may be less equipped to handle co‐morbidities in a population that previously had limitations preventing survival into adulthood. Adults with DS are at a higher risk for mental health disorders, as well as physical health problems such as thyroid disease, hearing impairments, osteoporosis, sleep apnea, congenital cardiac anomalies, dementia, and obesity (Capone et al., 2018; Foley et al., 2016; Rubenstein et al., 2020). Until recently, people with DS were mainly cared for by pediatric care providers because of the limited age span of the population (Tsou et al., 2020). As more patients survive into adulthood, more will need to transition from pediatric care providers to adult care providers.

Minimal guidance exists on how to undergo the process of transitioning care for people with DS. However, transition guidelines were offered in 2020 for those within the general population for different types of healthcare transitions (HCTs). These include transitioning youth to an adult provider and transitioning healthcare to an adult approach from a pediatric approach without changing clinicians (GotTransition.org, 2021). The six key steps in transitioning youth to an adult care provider are: (1) educating staff while discussing the transition process with patients and their families; (2) tracking and monitoring progress; (3) discussing gaps and needs; (4) regularly updating the plan of care, and consenting for release of medical information; (5) preparing medical records and final transition documents with the adult care provider; and (6) completion of the transfer, with confirmation of adult caregiver appointments and having additional consultations, as required (GotTransition.org, 2021).

While these transition guidelines may be useful for certain populations, they are not adequate for patients with special needs. Systematic reviews on youth with special healthcare needs (YSHCN), specifically those with congenital heart disease (Heery et al., 2015), cystic fibrosis (Coyne et al., 2017; Heery et al., 2015), and diabetes (Findley et al., 2015), have shown gaps in ensuring proper HCTs for those with these, and other complex health conditions (McManus et al., 2013). Presently, patients with DS may transition some, but not all, of their providers to those focused on adult care, or they may continue to solely see their pediatric care team (Gray et al., 2017). Of pertinence, patients who choose to remain with their pediatric care providers may experience a subpar quality of care due to a lack of training with certain co‐morbidities (Baumer & Davidson, 2014; Gray et al., 2017). For example, patients with DS are more likely to have differences in the presentation of certain disorders, such as depression, and therefore should receive care from providers specifically trained in working with individuals with DS (Baumer & Davidson, 2014).

Despite the lack of guidelines for assisting people with DS in navigating the HCT process and finding appropriate healthcare providers, to the best of our knowledge there has not been a review published that has examined the details of, and barriers to, HCT for those with DS. To better understand existing transition processes and to identify the groundwork for developing a guide to HCT for those living with DS, we aimed to provide a scoping review of the literature on the topic. Our aims in this review were threefold: (1) identify original research articles in peer‐reviewed medical journals that focus on key questions about transitioning from pediatric to adult healthcare providers for adults with DS; (2) evaluate the quality of the existing evidence, identify deficiencies in current clinical knowledge, and suggest directions for future research; and (3) begin to formulate practical guidelines to support best medical practices for transitioning adults with DS from pediatric to adult healthcare providers.

## 2 METHODS

### 2.1 Data sources

This review followed PRISMA‐ScR (Preferred Reporting Items for Systematic Reviews and Meta‐Analyses for Scoping Reviews) guidelines for scoping reviews (Page et al., 2021). On November 22, 2021, searches were conducted in PubMed, Scopus, and CINAHL to identify peer‐reviewed studies relating to patients with DS undergoing transitions of care. Our search terms were selected to be inclusive of patients with DS and the transference of care from pediatric care to adult care providers. Full search terms for all databases are available in Table 1. There were no restrictions on the date of publication.

**TABLE 1:** Complete search terms by database

PubMed	Scopus	CINAHL
(“Down Syndrome”[Mesh] OR “Trisomy 21”) **AND** (“Transition to Adult Care”[Mesh] or “Transitional Care”[Mesh] OR “Continuity of Care” OR “Health Services Accessibility”[Mesh] OR “Adult care” OR “Transition to adulthood” OR “Transition Care”)	(TITLE‐ABS‐KEY ({Down Syndrome}) OR TITLE‐ABS‐KEY ({Trisomy 21})) **AND** (TITLE‐ABS‐KEY ({Transition to Adult Care}) OR TITLE‐ABS‐KEY ({Transitional Care}) OR TITLE‐ABS‐KEY ({Continuity of Care}) OR TITLE‐ABS‐KEY ({Health Services Accessibility}) OR TITLE‐ABS‐KEY ({Adult care}) OR TITLE‐ABS‐KEY ({Transition to adulthood}) OR TITLE‐ABS‐KEY ({Transition Care}))	([MM “Down Syndrome”] OR “TRISOMY 21”) **AND** (“Transition to Adult care” OR (MM “Transitional Care”) OR “Continuity of care” OR (MM “Health Services Accessibility+”) OR (MM “Adult Care [Saba CCC]”) OR (MM “Transition to Adulthood”) OR (MM “Continuity of Patient Care+”))

Two authors (KV and RI) independently screened articles by title and abstract to determine eligibility for the review. The remaining articles were further assessed for full‐text review based on the inclusion and exclusion criteria to determine articles for the final analysis and hence data extraction. Articles were selected by the following inclusion criteria: written in English, based on original research (quantitative or qualitative), and included at least five patients with DS undergoing transitional care or planning for transitional care. This means that patients had to have either begun the process of transitioning or have started formal planning and discussions for the HCT process. Studies from any country were eligible for inclusion. We excluded reviews, commentaries, editorials, case reports, and case series with fewer than five patients. Furthermore, any article was excluded that analyzed patients with DS alongside other populations, such as patients with other neurological diseases, while undergoing a transition from pediatric to adult care, if the article did not provide a stratified analysis strictly for patients with DS.

### 2.2 Data extraction

Following an existing approach (Doug et al., 2011) from a review on transitional care for patients undergoing palliative care, the following information was extracted from each study: author and year of publication; country; research focus; design and methods; population; key findings; and comments/implications. Thereafter, data that answered the following five questions were extracted: (1) Are youth/young adults with DS more likely to have a gap in transition services? (2) What are common difficulties associated with HCT in people with DS? (3) How does a diagnosis of DS and severity of comorbid conditions affect HCT? (4) What patient‐specific factors negatively impact the transition of care? These data were independently extracted and then discussed within the group.

Each article underwent a quality assessment using a validated scoring system designed for studies with varied methodologies (Hawker et al., 2002). Each study could receive up to a total score of 32, based on eight measures with a potential value of 1–4. The eight measures assessed for each study were introduction and aims; method and data; sampling; data analysis; ethics and bias; findings/results; transferability/generalizability; and implications and usefulness. Two authors (KV and RI) independently assessed all included articles; discrepancies in scoring were resolved based on consensus between KV and RI.

## 3 RESULTS

### 3.1 Searches and articles included

**FIGURE 1:**
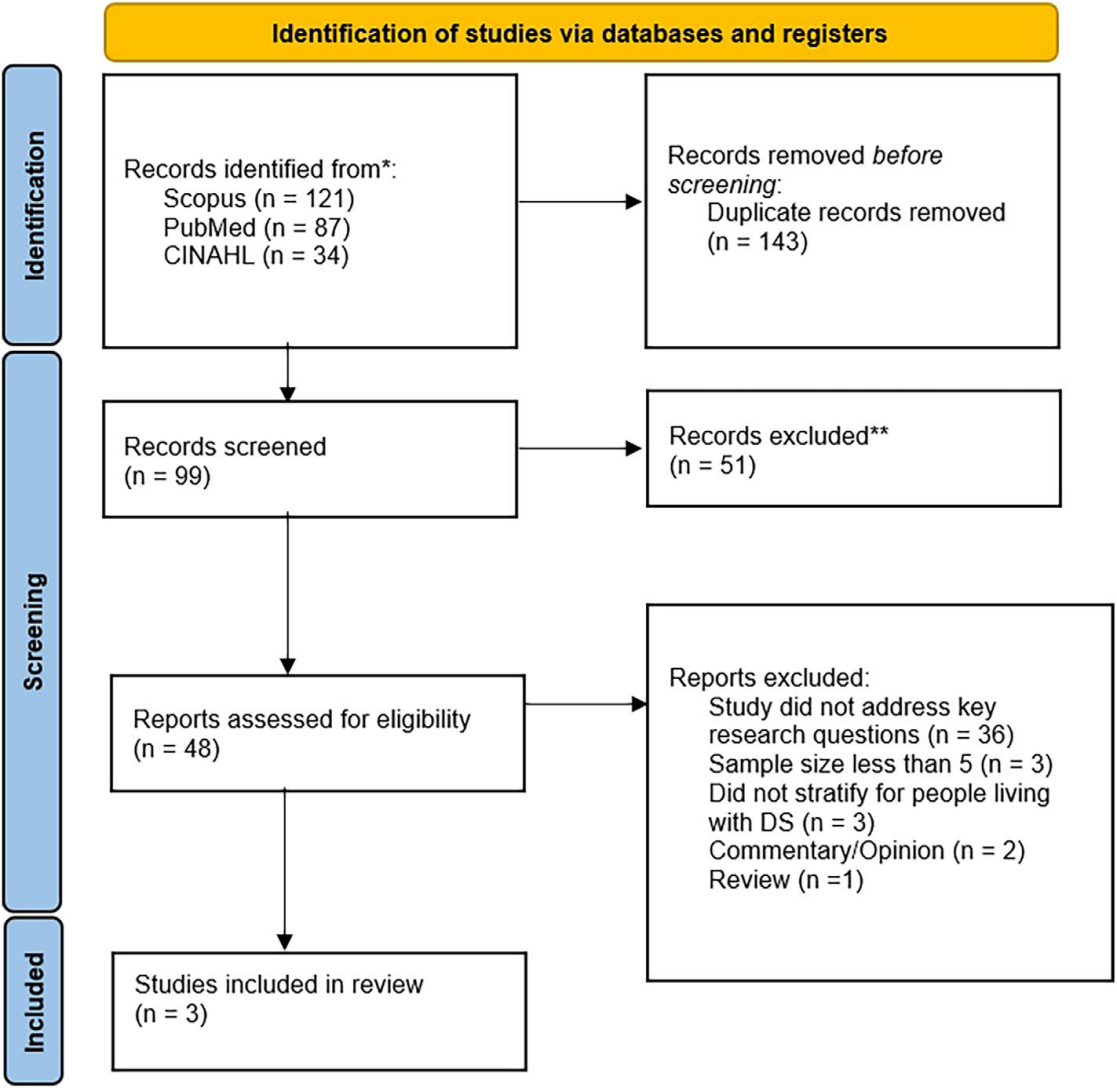
PRISMA‐ScR study selection flow diagram. *Source*: Page et al. (2021))

The initial searches produced 242 articles. A total of 143 duplicate articles were removed, and an additional 51 articles were removed after screening by title and abstract. Of the 48 articles that were assessed for full‐text eligibility, three articles met the selection criteria for full‐text data extraction (Figure 1).

### 3.2 Characteristics of selected articles

Data extracted and information on study characteristics are listed in Table 2. One study from 2013 utilized a retrospective cohort design to focus on individuals living with DS in Michigan (Jensen & Davis, 2013). The second, from 2014, also involved a retrospective analysis of patients in Texas at a facility that included patients with DS but also patients with other comorbidities (Berens & Peacock, 2014). The third study from 2018 involved the usage of a cross‐sectional design, via a survey, and included individuals living with DS from across the United States (Nugent et al., 2018). The article by Nugent et al. was deemed to be of the highest quality (with an assessment score of 31 out of 32), whereas the other studies were considered to be relatively high quality, despite not having as high a ranking for quality; two studies had high transferability/generalizability (Jensen & Davis, 2013; Nugent et al., 2018), whereas one only had fair transferability/generalizability (Berens & Peacock, 2014).

**TABLE 2:** Data extraction table, with study characteristics and findings

Author & year of publication	Jensen and Davis (2013)	Berens and Peacock (2014)	Nugent et al. (2018)
Location	Michigan, United States	Texas, United States	Across the United States
Research focus	To characterize healthcare patterns of adults with DS based on whether they had fully transitioned to adult‐oriented healthcare providers.	To describe the development and implementation of a pediatric to adult transition medical clinic for individuals with chronic childhood conditions.	To compare the prevalence of successful transition planning for youth with DS and youth with other special healthcare needs (OSHCN), and analyze the effects of different demographic and social factors on preparation for transition.
Design and methods	Retrospective observational cohort design, with health records of a single academic health center for 18‐ to 45‐year‐old DS patients receiving care between 2000 and 2008; healthcare utilization and annualized patient charges were evaluated.	Retrospective analysis of health records for patients receiving care before July 2011 in the Transition Medicine Clinic (TMC) in Houston Texas.	All data from 2009 to 2010 National Survey of Children with Special Health Care Needs (NS‐CSHCN) a random survey given across the United States. Respondents were either parents or guardians of children in the household. Transition core outcome is based on 4 individual measures: (i) shifting to an adult provider, (ii) adult healthcare needs, (iii) health insurance coverage maintenance, and (iv) more responsibility for self‐care. Ten variables associated with transition planning were controlled for.
Population	205 DS patients aged 18–45 were included.	332 patients of the TMC from 22 different Texas counties and 2 Louisiana counties. 54 patients (17%) had DS. At baseline, no patients at the TMC were receiving primary care from an adult‐focused provider and were either receiving care from a pediatrician or had been without medical care.	Of the 196,159 households that responded to the survey, 17,114 children ages 12–17 were included in the analysis: 151 had DS and 16,963 had OSHCN.
Key findings	52% of DS patients had incompletely transitioned to full adult care and had been seen by a combination of adult‐ and child‐focused providers, compared to 48% of patients who were only receiving care from adult‐focused providers. Both groups had similar proportions of hypothyroidism and atlanto‐axial instability, but 76% in the mixed provider group had congenital heart disease, compared to 9% in the adult provider group; 62% in the mixed care group had moderate or severe disease, compared to 6% in the adult care group. Both groups had similar levels of healthcare utilization, admissions to the hospital, and time in the intensive care unit. After controlling for covariates, the cost for mixed care was considerably higher, both with and without hospitalized visits; adult care patients' total charges were $2305 without hospitalization and $19,240 with hospitalization, compared to $2876 for mixed care without hospitalization, and $38,301 with hospitalization.	33.3% of DS patients had Medicaid only, 9.3% had Medicare only, 13.0% had private insurance only, and 44.5% had some combination. Mean age of the first TMC visit for DS patients was 27.3. 38.9% of DS patients had secondary morbidity of Vitamin D deficiency, 9.3% had osteopenia/osteoporosis, 18.5% had gastroesophageal reflux disease, 22.2% had constipation, 7.4% had seizures, 59.3% had obesity, 48.1% had sleep apnea, 37% had thyroid disease, and 46.3% had heart disease. Of those who did not have sleep apnea diagnosed, 82.1% had clinical symptoms of the disease in the electronic health records. The most common specialists utilized by transition medicine DS patients were cardiologists (64.8%), pulmonologists (63.0%), mental health providers (24.1%), endocrinologists (22.2%), and gastroenterologists.	More youth with DS (87.2%) had some kind of functional limitation compared to youth with OSHCN (22.9%), *p* < 0.001, and more comorbid conditions (*p* < 0.001). More youth with OSHCN (56.8%) had entirely private insurance coverage compared to youth with DS (35.3%), *p* < 0.001. 31.5% of youth with DS received care within a medical home, compared to 43.2% for youth with OSHCN (p = 0.075). 11% of youth with DS had met the transition core outcome, compared to 40% of youth with OSHCN (*p* < 0.001). Youth with DS were close to four times more likely to not receive healthcare transition planning compared to youth with OSHCN (OR: 3.99; 95% CI: 1.66–9.57). Youth with DS had 4.24 times higher odds of not being encouraged to take responsibility for their care compared to youth with OSHCN (OR: 4.24; 95% CI: 2.14–8.42). No significant association between DS and other three individual outcome component measures (*p* > 0.05 for: did not discuss the shift to an adult provider, did not discuss adult healthcare needs, did not discuss health insurance).
Comments and implications	Those with mixed care tend to have higher costs, and higher proportions and complexities for congenital heart disease. Findings suggest that reasons for adults referred to child‐focused providers in the clinic are connected to the need for medically intensive care.	Familiarity with multiple insurance types and structures is needed for providing care to the TMC population. It is important for physicians to recognize how to manage secondary diagnoses, and, as TMC patients saw an average of 3.8 specialists, it is important for providers to be connected with relevant specialists. Sleep apnea was particularly high among DS patients, and it is recommended that they be screened throughout the lifespan, particularly as they reach adulthood. Under current models of reimbursement, not financially sustainable; a need to demonstrate improved care and savings from the limited hospital and emergency visits.	As a youth with DS had low transition success, regardless of the number of screening criteria qualified, it is suggested that factors other than disease severity are causing the disparity in transition planning. Disparities in transition planning, unmet healthcare needs, delayed care, and financial stress may be reduced by the presence of a medical home for youth with DS.
Quality assessment score	30	28	31

### 3.3 Population under consideration

All three studies included populations in the United States. While one study focused solely on patients with DS without controls (Jensen & Davis, 2013), the other two studies included a comparison of patients with DS undergoing HCT either with those living with a chronic disease (Berens & Peacock, 2014) or children with other special healthcare needs (OSHCN) (Nugent et al., 2018). More precisely, in Berens and Peacock (2014), comparisons were made between patients with DS and those with an array of other health conditions, such as cerebral palsy, spina bifida, and autism.

Study samples included 205 patients with DS aged 18–45 (Jensen & Davis, 2013); 54 patients with DS (out of 332 total patients with various health conditions) with a mean age of 27.3 years (Berens & Peacock, 2014); and 151 with DS (out of 17,114 patients) between the age of 12 and 17 (Nugent et al., 2018).

### 3.4 Key questions

Data extracted from key questions are listed in Table 3.

**TABLE 3:** Data extraction from key questions

Study	Jensen and Davis (2013)	Berens and Peacock (2014)	Nugent et al. (2018)
Likelihood of gap in transition services	Probable, the majority of adults with DS had incompletely transitioned.	At baseline, no patients at the TMC were receiving primary care from an adult‐focused provider and were either receiving care from a pediatrician or had been without medical care.	Only 11% of adolescents with DS met transition core outcomes, compared to 40% of adolescents with OSHCN.
Difficulties in clinical transition	A similar pattern of healthcare utilization between patients in both provider groups.	Patients with DS had an especially high prevalence of sleep apnea and the majority received care from cardiologists and pulmonologists.	Adolescents with DS were less likely to be encouraged to take responsibility for their health than adolescents with.
Impact of diagnoses	Patients seeing child‐focused providers had increased complexity of CHD compared to peers with an only adult‐focused providers.	Not discussed.	Diagnosis of DS is associated with more comorbid conditions than adolescents with OSHCN.
Patient‐specific factors	Increased complexity or severity of illness. Increased financial cost to those with mixed care providers, only in the case of hospitalization.	Sleep apnea, heart disease, and associated complications, obesity, and thyroid disease. Outside funding to the TMC is required due to the greater number of clinical resources required.	Male sex, Black or Hispanic race, poverty, lack of insurance coverage, public insurance, functional limitations. Those who have private insurance or come from a medical home may be more likely to receive transition services.

#### 3.4.1 Key question #1: Likelihood of care gaps during transition

In all three of the studies included, patients with DS consistently experienced incomplete HCT. In Jensen and Davis' (2013) article, 52% of patients with DS had incomplete transitions. In the study by Nugent et al. (2018), only 11% of patients actually met transition core outcomes, compared to 40% of youth with OSHCN who met these outcomes. Furthermore, youth with DS had 3.99 times higher odds of not receiving transition planning compared with youth with OSHCN (Nugent et al., 2018). In the Berens and Peacock (2014) study, none of the patients had been receiving primary care from an adult provider at baseline.

#### 3.4.2 Key question #2: Difficulties in clinical transition

Both studies which included comparison groups (Berens & Peacock, 2014; Nugent et al., 2018) found similarities between the two groups; however, in the Nugent et al. (2018) study, youth with DS had 4.24 times higher odds of not being encouraged to take responsibility for their own care compared with the youth of OS. In the article by Berens and Peacock (2014), individuals with DS were requiring additional care from cardiologists and/or pulmonologists and were found to have high rates of sleep apnea.

#### 3.4.3 Key question #3: Impact of diagnoses

In one study, among patients only seeing pediatric care providers, around 76% of patients tended to have increased complexities of chronic heart disease (CHD), compared to only 9% of those who had been receiving care from adult providers (Jensen & Davis, 2015). Furthermore, Jensen and Davis (2015) showed that 62% of patients in the mixed care group had moderate or severe disease, compared to the 6% of patients who received adult‐specific care. Berens and Peacock (2014) showed that patients with DS had higher rates of obesity (59.3%), and sleep apnea (48.1%) compared to those with autism, cerebral palsy, spina bifida, and those who were fragile or had some other genetic condition. Also, patients with DS in this study had relatively high rates of Vitamin D deficiency (38.9%), constipation (22.2%), and gastroesophageal reflux disease (18.5%), though their rates were lower than those with other medical conditions. Another study found that a diagnosis of DS was associated with a higher number of health conditions compared to those with OSHCN, though whether or not this was related to their HCT is not clear (Nugent et al., 2018).

#### 3.4.4 Key question #4: Patient‐specific factors

An increase in the prevalence of comorbidities and increased severity of disease presentation were high‐risk indicators in two studies; these conditions included sleep apnea (48.1%), CHD (46.3%), gastroesophageal reflux disease (18.5%), obesity (59.3%), and thyroid disease (37%) (Berens & Peacock, 2014; Jensen & Davis, 2013). Nugent et al. (2018) focused on demographic factors, and found the following factors to increase the odds of not receiving transitional planning services: being male (1.38 times higher compared to females), being Black or Hispanic (1.49 and 1.56 times higher, respectively, compared to White adolescents), living in poverty (1.26 times higher compared to adolescents at 400% or above the federal poverty line), lack of medical home (2.36 odds compared to those with a medical home), and limited/public insurance (public insurance alone carries a 1.71 higher odds than private insurance, and uninsured adolescents have 2.36 times higher odds).

In one of the three studies included, findings showed that those who either came from a medical home or who had private insurance had higher odds of receiving transition services (Nugent et al., 2018). The two other studies found that greater funding was required for care providers due to the need for more complex services; in the study of Jensen and Davis (2013), this was seen clearly in cases that resulted in hospitalization, as adult care patients' total charges were $19,240 compared to the $38,301 charged to patients who received mixed care in the hospital.

## 4 DISCUSSION

The findings of this review indicate that patients with DS consistently tended to have incomplete HCTs from pediatric to adult care providers and receive little or no transitional care planning. A significant proportion of patients had not undergone any transition at all. Given that the lifespan of individuals with DS has increased dramatically over the past decades, it is not unsurprising that the adult medical system is not as well prepared to provide them with comprehensive care.

The findings of our review also show that financial barriers contribute to the incomplete HCT for patients with DS, and these have been shown to be linked to insurance issues or limited time/resources among the providers. Consistent with the joint transition statement of the American Academy of Pediatrics, American Academy of Family Physicians, and the American College of Physicians (White & Cooley, 2018), these findings highlight that increased funding will be needed for public health insurance to ensure that patients will be eligible for transitional care and that care providers are properly reimbursed for offering these services, along with modifications to existing policies.

Patients with DS consistently were found to have a high proportion of risk factors and comorbidities when compared to those with other health conditions and OSHCN. Those with DS who did not undergo complete transitional care were those who tended to have more severe and complex symptoms of the comorbid conditions. This further emphasizes the need to increase funding and resources to ensure proper transitional care for this population; sufficient and coordinated HCTs are likely to reduce costs over the long term as patients will be more likely to have appropriate screenings and care.

The first evidence‐based guidelines for the care of adults with DS were only recently published, and while they cover critical topics related to adults with DS, they do not address the transition to adult care and only cover a limited number of conditions (Tsou et al., 2020). The American Academy of Pediatrics guidelines for children with DS (Bull, 2011) do recommend a transition to adulthood be addressed as part of anticipatory guidance during the adolescent years but do not provide a suggested framework. There are limited numbers of specialized clinics providing primary and consultative care to adults with DS, and they may be an important resource to patients and healthcare providers with less experience caring for adults with DS but are not accessible to all. Advocacy organizations, local DS associations, or other organizations that serve adults with intellectual disabilities may be valuable resources for clinicians, patients, and caregivers. Examples of such organizations, along with a brief description of their stated objectives, are listed in Table 4.

**TABLE 4:** Organizations that support and advocate for people living with DS

Organization	Summarized objectives of organization
Down Syndrome Medical Interest Group – USA (DSMIG‐USA)	Professionals and families focus on improving the optimal care and well‐being of individuals with DS of all ages (DSMIG‐USA, 2022).
Global Down Syndrome Foundation (GLOBAL)	Part of a network of organizations that work to improve the quality of life for those with DS by focusing on the areas of healthcare, advocacy, research, and education (Global Down Syndrome, 2022).
Down Syndrome Federation of India (DSFI)	Offering support to individuals with DS and their families through the offering of support services, advocacy, and counseling for families (DSFI, 2022).
National Down Syndrome Congress (NDSC)	Improving the world for those with DS, offering support and information for those affected by DS/wanting to learn about DS (NDSC, 2022).
European Down Syndrome Association (EDSA)	To ensure development in every aspect of life for people with DS by connecting organizations, sharing information, and establishing collaborations (EDSA, 2022).
National Down Syndrome Society (NDSS)	A human rights organization focused on improving the quality of life of those with DS with policy & advocacy, community engagement, and resources & support (NDSS, 2022).
Down Syndrome Australia	Support people living with DS and their families primarily by influencing social and public policy (Down Syndrome Australia, 2022).

**FIGURE 2:**
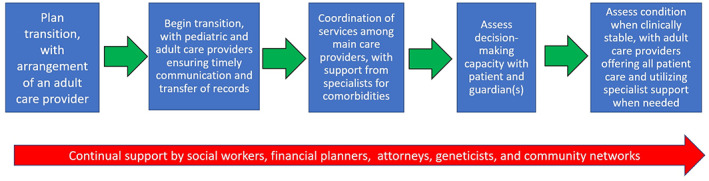
Proposed transition guidelines from pediatric care providers to adult care providers for people living with DS

Based on our findings, we have proposed potential steps of transition for a patient with DS in Figure 2. Finding an adult primary care provider willing to care for the adult with DS is a logical first step in transitioning to an adult model of care. Real barriers to finding such providers exist, however, and this step may take time (Prokup et al., 2017). A warm handoff from a long‐time pediatric specialty or primary care provider may open doors, and be an opportunity to help educate the adult provider. As systems of care and scope of practice may differ in the adult models of care, especially in patients who are clinically stable, the number of specialty providers needed to care for the patient may decrease. For example, the adult provider may be able to manage thyroid disease and menstruation. Unfortunately, some services, such as the care for patients with complex congenital heart disease, may not exist in typical adult healthcare systems ‐ meaning that some patients will need to continue to receive care in both pediatric and adult systems. It is important in these cases for the primary care provider and specialty provider to ensure timely transfer of records back and forth to one another and develop contingency planning and lines of communication in the event that the patient has to be admitted to either a pediatric or adult system that is not able to meet all their healthcare needs.

Decision‐making capacity should be evaluated at the time YSHCN approach the age of 18, when individuals reach the age of majority, and periodically throughout the lifespan. Specific tools exist to help clinicians evaluate decision‐making capacity for individuals with intellectual and developmental disabilities (Vanderbilt Kennedy Center, 2022), and a range of options exist to support youth transitioning to adulthood. While traditionally, guardianship was felt to be the best option for individuals with intellectual disability, less restrictive options such as power of attorney and supported decision‐making may be more appropriate (National Disability Rights Network, 2019).

As individuals with DS may continue to reside in the family home, it is not only important to address decision‐making and incorporate the patient's beliefs and wishes into their care but to also ensure that other individuals who may help care for the person with DS when parents are unable to are aware of their medical history and care team. To the extent possible, discussions involving the person with DS need to take place around succession planning whether guardianship, power of attorney, or supported decision‐making is in place. Questions to consider also include where will the individual live and in what setting when they can no longer live at home. If their new living situation will be in another state, attention needs to be paid to the impact on the individual in terms of impact on support services, friendships, and community networks along with access to appropriate medical services. Involving a team including a social worker, attorney, and financial planner may be beneficial.

Limitations of this review need to be considered. A small number of studies were ultimately eligible for this scoping review, therefore limitations in attempting to draw conclusions exist. The current gaps in data therefore suggest a clear need for more research on HCT for patients with DS to be conducted. Furthermore, though studies from any region were eligible for inclusion, all studies that were included in the analysis were quantitative studies conducted in the United States; hence, a need exists for more studies in different regions with different cultural contexts.

Notably, this review provides important insights for those with other genetic conditions. As DS is the most common genetic cause of intellectual disability, it can potentially serve as a model for completely transitioning other youth with genetic syndromes to adult models of care. As patients with DS and significant congenital heart disease may continue to receive some care in predominantly pediatric systems of care, the geneticist can become the expert or liaison to adult systems for their patients who need to continue to receive care in both systems. Furthermore, our work provides a clear insight into gaps that are suitable for future research, and the broad nature of the research questions allowed for a thorough analysis of circumstances faced by patients with DS who are undergoing HCT. It will be important for future research to evaluate the extent to which patients with DS are, or are not, transitioning to an adult care provider who is able to address their complex health needs. It will also be highly useful for future research to focus on aspects of HCT that can improve outcomes for specific health conditions that individuals with DS are more likely to face. As people with DS have an increased susceptibility to dementia, obstructive sleep apnea, osteoporosis, and various other health problems (Capone et al., 2018; Foley et al., 2016; Nugent et al., 2018; Rubenstein et al., 2020), it would be important to understand how HCTs to appropriate care providers may reduce the symptomatic presentation of these comorbidities.

Our findings showed that individuals with DS who are transitioning into adulthood currently face many difficulties and barriers that limit the extent to which they can smoothly transition from care by pediatric providers to adult providers. We therefore have proposed steps for transition for people with DS. Future research is needed surrounding HCTs to improve the evidence‐based guidelines that ultimately ensure that this demographic receives the care they need.

## AUTHOR CONTRIBUTIONS

Karan Varshney wrote most portions of the manuscript, conducted the full review process, extracted data, conducted quality assessments, participated in planning the project, coordinated the team, and made major edits and revisions to the manuscript. Rosemary Iriowen conducted the full review process, extracted data, and conducted quality assessments. Kayla Morrell and Preshon Pillay wrote portions of the manuscript and provided edits. Alexander Fossi and Mary M. Stephens participated in planning the project, provided expertise, and made major edits and revisions to the manuscript.

## CONFLICT OF INTEREST

The authors have no conflicts of interest to disclose.

## Data Availability

Data sharing not applicable to this article as no datasets were generated or analysed during the current study.

## References

[ajmga62854-bib-0001] Baumer, N. , & Davidson, E. J. (2014). Supporting a happy, healthy adolescence for young people with down syndrome and other intellectual disabilities: Recommendations for clinicians. Current Opinion in Pediatrics, 26(4), 428–434. 10.1097/MOP.0000000000000122 25010137

[ajmga62854-bib-0002] Berens, J. , & Peacock, C. (2014). Implementation of an academic adult primary care clinic for adolescents and young adults with complex, chronic childhood conditions. Journal of Pediatric Rehabilitation Medicine: An Interdisciplinary Approach, 8, 3–12. 10.3233/PRM-150313 25737343

[ajmga62854-bib-0003] Bittles, A. H. , & Glasson, E. J. (2004). Clinical, social, and ethical implications of changing life expectancy in down syndrome. Developmental Medicine and Child Neurology, 46(4), 282–286. 10.1111/j.1469-8749.2004.tb00483.x 15077706

[ajmga62854-bib-0004] Bull, M. J. (2011). Health supervision for children with down syndrome. Pediatrics, 128(2), 393–406. 21788214 10.1542/peds.2011-1605

[ajmga62854-bib-0005] Capone, G. T. , Chicoine, B. , Bulova, P. , Stephens, M. , Hart, S. , Crissman, B. , Videlefsky, A. , Myers, K. , Roizen, N. , Esbensen, A. , Peterson, M. , Santoro, S. , Woodward, J. , Martin, B. , & Smith, D. (2018). Co‐occurring medical conditions in adults with down syndrome: A systematic review toward the development of health care guidelines. American Journal of Medical Genetics. Part A, 176(1), 116–133. 10.1002/ajmg.a.38512 29130597

[ajmga62854-bib-0006] Center for Disease Control and Prevention . (2020). Data and statistics on down syndrome. CVC.gov. Retrieved from. https://www.cdc.gov/ncbddd/birthdefects/downsyndrome/data.html

[ajmga62854-bib-0007] Center for Disease Control and Prevention . (2021). National Vital Statistics System. CDC.gov. Retrieved from. https://www.cdc.gov/nchs/about/factsheets/factsheet_nvss.htm

[ajmga62854-bib-0008] Coyne, I. , Sheehan, A. M. , Heery, E. , & While, A. E. (2017). Improving transition to adult healthcare for young people with cystic fibrosis: A systematic review. Journal of Child Health Care, 21(3), 312–330. 10.1177/1367493517712479 29119815

[ajmga62854-bib-0009] de Graaf, G. , Buckley, F. , & Skotko, B. G. (2017). Estimation of the number of people with down syndrome in the United States. Genetics in Medicine, 19(4), 439–447. 10.1038/gim.2016.127 27608174

[ajmga62854-bib-0010] Deng, C. , Yi, L. , Mu, Y. , Zhu, J. , Qin, Y. , Fan, X. , Wang, Y. , Li, Q. , & Dai, L. (2015). Recent trends in the birth prevalence of down syndrome in China: Impact of prenatal diagnosis and subsequent terminations. Prenatal Diagnosis, 35(4), 311–318. 25315427 10.1002/pd.4516

[ajmga62854-bib-0011] Doidge, J. C. , Morris, J. K. , Harron, K. L. , Stevens, S. , & Gilbert, R. (2020). Prevalence of Down's syndrome in England, 1998–2013: Comparison of linked surveillance data and electronic health records. International Journal of Population Data Science, 5(1), 1157. 32864476 10.23889/ijpds.v5i1.1157 PMC7115985

[ajmga62854-bib-0012] Doug, M. , Adi, Y. , Williams, J. , Paul, M. , Kelly, D. , Petchey, R. , & Carter, Y. H. (2011). Transition to adult services for children and young people with palliative care needs: A systematic review. Archives of Disease in Childhood, 96(1), 78–84. 10.1136/adc.2009.163931 19948663

[ajmga62854-bib-0013] Down Syndrome Australia . (2022). Retrieved from https://www.downsyndrome.org.au/national/.

[ajmga62854-bib-0014] Down Syndrome Federation of India (DSFI) . (2022). Retrieved from https://downsyndrome.in/.

[ajmga62854-bib-0015] Down Syndrome Medical Interest Group–USA (DSMIG‐USA) . (2022). Retrieved from https://www.dsmig-usa.org/.

[ajmga62854-bib-0016] Education Down Syndrome Association. EDSA . (2022). Retrieved from http://www.edsa.eu/.

[ajmga62854-bib-0017] Findley, M. K. , Cha, E. , Wong, E. , & Faulkner, M. S. (2015). A systematic review of transitional Care for Emerging Adults with diabetes. Journal of Pediatric Nursing, 30(5), e47–e62. 10.1016/j.pedn.2015.05.019 26164412 PMC4567467

[ajmga62854-bib-0018] Foley, K. , Taffe, J. , Bourke, J. , Einfeld, S. L. , Tonge, B. J. , Trollor, J. , & Leonard, H. (2016). Young people with intellectual disability transitioning to adulthood: Do behaviour trajectories differ in those with and without down syndrome? PLoS One, 11(7), e0157667. 10.1371/journal.pone.0157667 27391326 PMC4938609

[ajmga62854-bib-0019] Global Down Syndrome Foundation (GDSA) . (2022) Retrieved from https://www.globaldownsyndrome.org/.

[ajmga62854-bib-0020] GotTransition.org . (2021). Six core elements of health care transition. GotTransition.org.

[ajmga62854-bib-0021] Gray, W. N. , Schaefer, M. R. , Resmini‐Rawlinson, A. , & Wagoner, S. T. (2017). Barriers to transition from pediatric to adult care: A systematic review. Journal of Pediatric Psychology, 43(5), 488–502. 10.1093/jpepsy/jsx142 29190360

[ajmga62854-bib-0022] Hawker, S. , Payne, S. , Kerr, C. , Hardey, M. , & Powell, J. (2002). Appraising the evidence: Reviewing disparate data systematically. Qualitative Health Research, 12(9), 1284–1299. 10.1177/1049732302238251 12448672

[ajmga62854-bib-0023] Heery, E. , Sheehan, A. M. , While, A. E. , & Coyne, I. (2015). Experiences and outcomes of transition from pediatric to adult health Care Services for Young People with congenital heart disease: A systematic review. Congenital Heart Disease, 10(5), 413–427. 10.1111/chd.12251 25659600

[ajmga62854-bib-0024] Jensen, K. M. , & Davis, M. M. (2013). Health care in adults with down syndrome: A longitudinal cohort study: Health care in adults with down syndrome. Journal of Intellectual Disability Research, 57(10), 947–958. 10.1111/j.1365-2788.2012.01589.x 22775057

[ajmga62854-bib-0025] McManus, M. A. , Pollack, L. R. , Cooley, W. C. , Mcallister, J. W. , Lotstein, D. , Strickland, B. , & Mann, M. Y. (2013). Current status of transition preparation among youth with special needs in the United States. Pediatrics, 131(6), 1090–1097. 10.1542/peds.2012-3050 23669518

[ajmga62854-bib-0026] National Disability Rights Network . (2019). Supported Decision Making and Health Care. Retrieved from https://www.ndrn.org/resource/supported‐decision‐making‐and‐health‐care/#:~:text=Supported%20decision%20making%20allows%20athe%20assistance%20the%20person%20needs.&text=Persons%20with%20disabilities%20can%20use,or%20she%20deems%20is%20needed.

[ajmga62854-bib-0027] National Down Syndrome Congress (NDSC) . (2022). Retrieved from https://www.ndsccenter.org/.

[ajmga62854-bib-0028] National Down Syndrome Society (NDSS) . (2022). Retrieved from https://www.ndss.org/ .

[ajmga62854-bib-0029] Nugent, J. , Gorman, G. , & Erdie‐Lalena, C. R. (2018). Disparities in access to healthcare transition Services for Adolescents with down syndrome. The Journal of Pediatrics, 197, 214–220. 10.1016/j.jpeds.2018.01.072 29571933

[ajmga62854-bib-0030] Page, M. J. , McKenzie, J. E. , Bossuyt, P. M. , Boutron, I. , Hoffmann, T. C. , Mulrow, C. D. , Shamseer, L. , Tetzlaff, J. M. , Akl, E. A. , Brennan, S. E. , Chou, R. , Glanville, J. , Grimshaw, J. M. , Hróbjartsson, A. , Lalu, M. M. , Li, T. , Loder, E. W. , Mayo‐Wilson, E. , McDonald, S. , … Moher, D. (2021). The PRISMA 2020 statement: An updated guideline for reporting systematic reviews. BMJ, 2021(372), n71. 10.1136/bmj.n71 PMC8005924 33782057

[ajmga62854-bib-0031] Prokup, J. A. , Andridge, R. , Havercamp, S. M. , & Yang, E. A. (2017). Health care disparities of Ohioans with developmental disabilities across the lifespan. Annals of Family Medicine, 15(5), 471–474. 10.1370/afm.2108 28893818 PMC5593731

[ajmga62854-bib-0032] Rubenstein, E. , Hartley, S. , & Bishop, L. (2020). Epidemiology of dementia and Alzheimer disease in individuals with down syndrome. JAMA Neurology, 77(2), 262–264. 10.1001/jamaneurol.2019.3666 31657825 PMC6820036

[ajmga62854-bib-0033] Tsou, A. Y. , Bulova, P. , Capone, G. , Chicoine, B. , Gelaro, B. , Harville, T. O. , Martin, B. A. , McGuire, D. E. , McKelvey, K. D. , Peterson, M. , Tyler, C. , Wells, M. , & Whitten, M. S. (2020). Medical Care of Adults with down syndrome: A clinical guideline. JAMA: The Journal of the American Medical Association, 324(15), 1543–1556. 10.1001/jama.2020.17024 33079159

[ajmga62854-bib-0034] United Nations (UN) . (2021). Down syndrome. United Nations.

[ajmga62854-bib-0035] Vanderbilt Kennedy Center . (2022). Health care for adults with intellectual and developmental disabilities–Toolkit for primary care providers. Vanderbilt Kennedy Center.

[ajmga62854-bib-0036] White, P. H. , & Cooley, W. C. (2018). Supporting the health care transition from adolescence to adulthood in the medical home. Pediatrics (Evanston). Pediatrics, 142(5), e20182587. 10.1542/peds.2018-2587 30348754

